# Supplementary material for: Experimental Selection of Paromomycin Resistance in Leishmania donovani Amastigotes Induces Variable Genomic Polymorphisms
Source: Microorganisms. 2021 Jul 21;9(8):1546. doi: 10.3390/microorganisms9081546 (PMC8398221; doi:10.3390/microorganisms9081546)
Supplement: Supplementary file 1 [file microorganisms-09-01546-s001.zip › microorganisms-1285507-supplementary.pdf]

Supplementary materials

Experimental Selection of Paromomycin Resistance in *Leishmania donovani* Amastigotes Induces Variable Genomic Polymorphisms

Sarah Hendrickx [1,†], João Luís Reis-Cunha [2,†], Sarah Forrester [2], Daniel C. Jeffares [2,*,‡] and Guy Caljon [1,*,‡]

[1] Laboratory of Microbiology, Parasitology and Hygiene (LMPH), University of Antwerp, 2610 Antwerp, Belgium; sarah.hendrickx@uantwerpen.be
[2] Department of Biology and York Biomedical Research Institute, University of York, York YO31 5DD, UK; jaumlrc@gmail.com (J.L.R.-C.); sarah.forrester@york.ac.uk (S.F.)
* Correspondence: daniel.jeffares@york.ac.uk (D.C.J.); guy.caljon@uantwerpen.be (G.C.); Tel.: +32-3-265-26-01 (G.C.)
† These authors contributed equally to this work.
‡ Share senior authorship.

Genomic DNA from promastigotes from twelve different *Leishmania* clones and the non-cloned population with varying paromomycin (PMM) susceptibility was used to generate paired-end libraries. Pooled and barcoded libraries were sequenced. Reads were mapped to the *L. donovani* BPK282A1 genome and detailed description of each read library are summarized in **Table S1**.

SNVs distribution and allele frequency variation among *L. donovani* isolates was used to estimate populational diversity and potential association of mutations with PMM resistance. The populational genetic diversity (π) is shown in **Figure S1**.

To evaluate the impact of gene and chromosomal copies alterations to PMM resistance, the copies of all *L. donovani* 8,135 genes and 36 chromosomes was estimated for the 13 sequenced isolates, and compared with their resistance profiles to PMM. Correlation between SNVs and PMM resistance in all *L. donovani* chromosomes is evaluated in **Figure S2** and the SNV correlated to PMM resistance are summarized in **Table S2**. In addition, the somy of each chromosome from each *L. donovani* isolate was estimated based in the median coverage of non-outlier genes. Chromosomal CNV among the *L. donovani* isolates is shown in **Figure S3** and the correlation between gene CNVs and PMM resistance in all *L. donovani* chromosomes is demonstrated in **Figure S4**.

Clone/strain	SRA ID	Reads	Mapped Reads	Mapped Reads %	Genome Coverage (x)	SNVs	SNPs	Indels
**Pre S57**	X	14514805	13214282	91.04	51	1766	1147	619
**CL1 S58**	X	14307223	12378659	86.52	52	2024	1203	821
**CL2 S59**	X	14508882	11783680	81.22	50	2019	1198	821
**CL3 S60**	X	12231707	11432053	93.46	57	2021	1201	820
**CL4 S61**	X	14869211	13365522	89.89	56	2024	1203	821
**CL5 S62**	X	13404877	11439010	85.33	48	2017	1200	817
**CL6 S63**	X	17981183	16235504	90.29	71	2022	1202	820
**CL7 S64**	X	15515187	13297788	85.71	54	2020	1200	820
**CL8 S65**	X	13342473	11465874	85.94	46	2022	1201	821
**CL9 S66**	X	14306265	13062978	91.31	57	2024	1203	821
**CL12 S68**	X	12024677	10229432	85.07	44	2022	1201	821
**CL13 S69**	X	12231707	11432053	93.46	47	2023	1202	821
**CL14 S70**	x	13079958	11793170	90.16	50	2019	1201	818

Table S1: WGS read libraries and SNV counts in the 12 clones and the polyclonal original population (Pre S57).

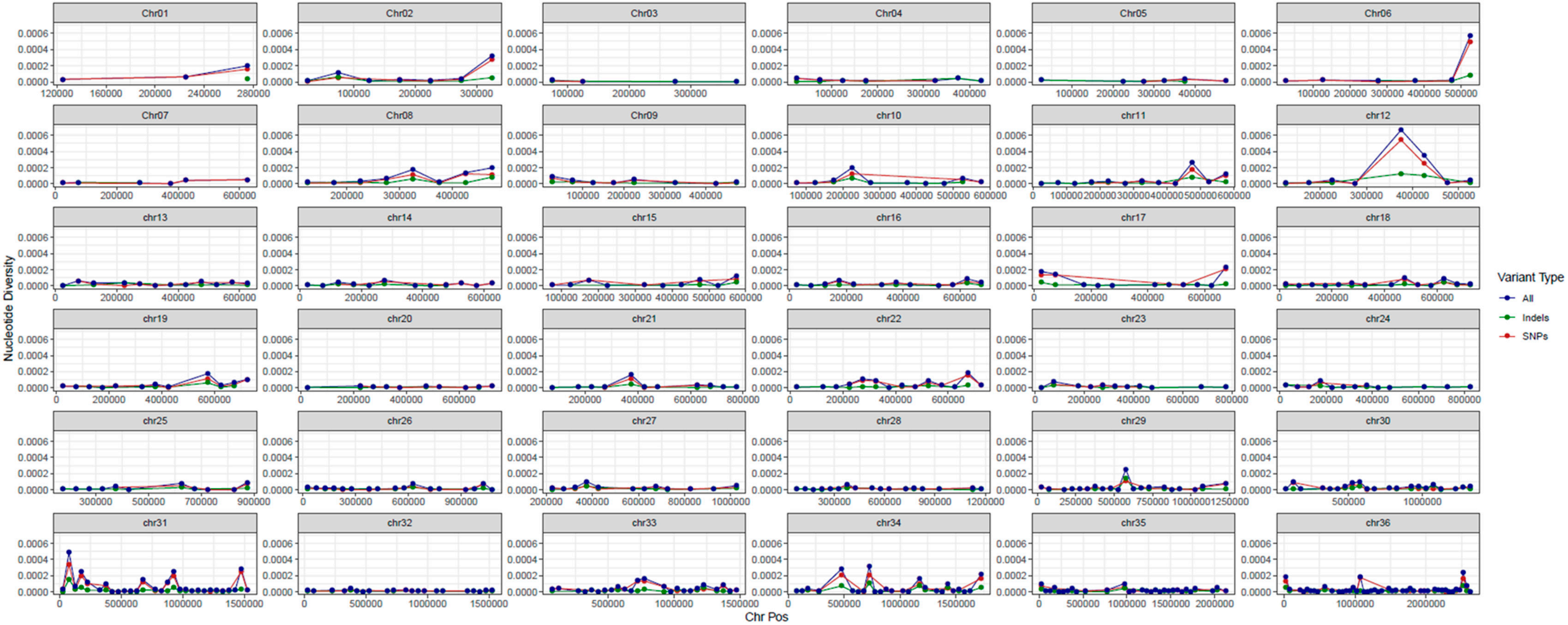

Figure S1: Genetic diversity (π) in the 36 *L. donovani* chromosomes. Populational genetic diversity (π) in chromosomes 1 – 36, estimated using 50 kb windows. The Y and X axis represent, respectively, genetic diversity and chromosomal position. SNPs, Indels and SNVs (All) are represented in green, blue and red, respectively.

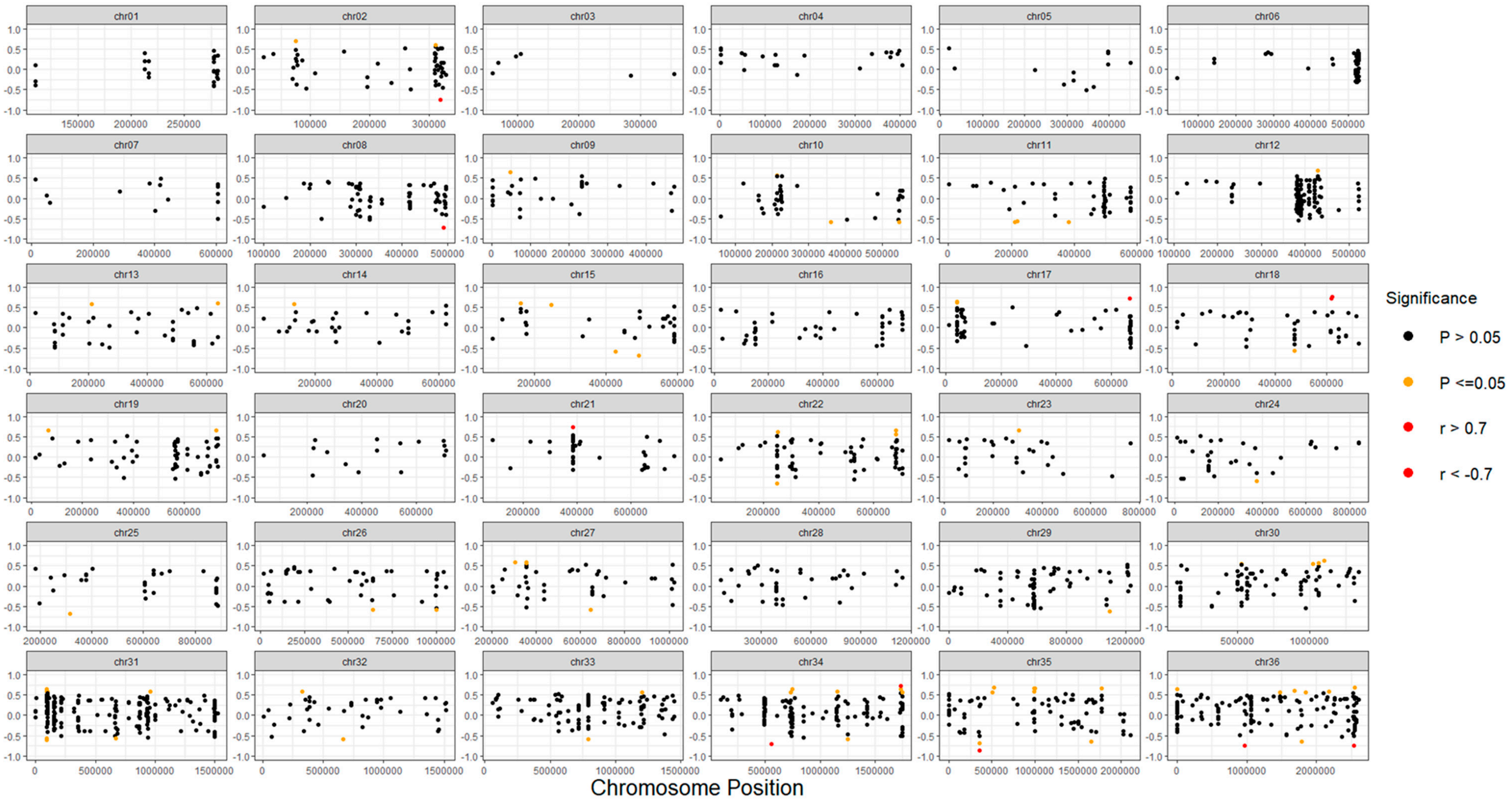

Figure S2: Correlation between SNVs and PMM resistance in all *L. donovani* chromosomes. Chromosomal distribution and correlation of SNVs allele frequency and PMM resistance. The X and Y axis correspond to respectively the chromosome position of each SNV and its correlation to PMM phenotype. Significant correlations are represented by orange dots. SNVs with strong significant positive or negative correlations (r ≥ 0.7 and r ≤ -0.7) are represented by red dots.

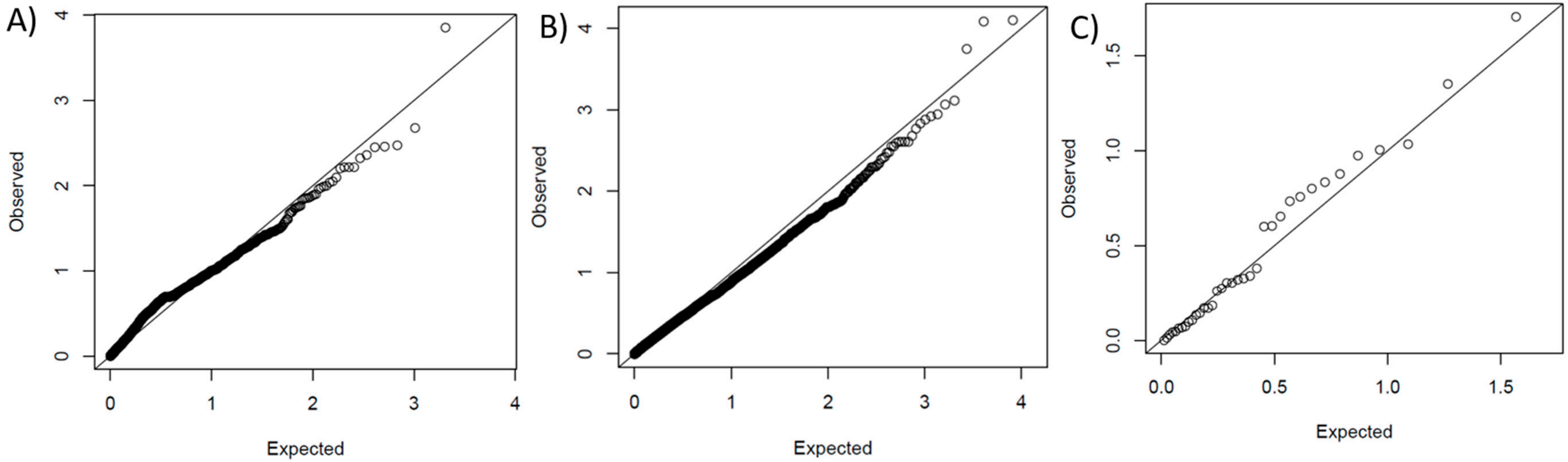

Figure S3. QQ plot of the genome variations association with phenotypes. Observed correlation -log10 P values are plotted against expected. A) Allele frequency fluctuations; B) Gene Copy Number Variation, C) Chromosome Copy Number Variation.

Table S2: SNV correlated to PMM resistance. This table summarizes the alleles, location and impact of SNVs potentially correlated with PMM resistance. Chr: chromosome number; Pos: chromosome position; REF: *L. donovani* genome reference allele; ALT: Alternate allele. Correlation: Pearson correlation between the alternate allele frequency and the PMM resistance; P: Correlation P-value; Max: Maximum alternate allele frequency in all clone/population; Min: Minimum alternate allele frequency in all clone/population; Difference: The difference between the Max and Min allele frequencies. Impact: The impact of the SNV.

Alternate Allele gain

Chr	Pos	REF	ALT	correlation	correlation_P	max	min	difference	Impact	Position_Change	Chromosome	NCBI ID	TriTrypDB ID	Annot
chr17	668944	G	A	0.7284	0.0047	0.7500	0.3176	0.4324	intergenic_region	n.668944G>A	LDBPK_171590-CHR_END			
chr18	618151	AGTGGGGGGGGTAA	AGTGGGGGGGGGTAA	0.7125	0.0063	0.6786	0.0323	0.6463	intergenic_region	n.618162_618163insG	LDBPK_181400-LDBPK_181410			
chr18	620025	CGTGTGTGTGTGTGTGTGTGTGC	CGTGTGTGTGTGTGTGTGTGC	0.7696	0.0021	0.7045	0.0571	0.6474	intergenic_region	n.620045_620046delTG	LDBPK_181400-LDBPK_181410			
chr21	384781	CTTC	GTCT	0.7331	0.0044	0.4188	0.2000	0.2188	intergenic_region	n.384781_384784delCTTCinsGTCT	LDBPK_211160-LDBPK_211170			
chr34	558711	AGGGGGGGGGTGAGAGC	AGGGGGGGGGGTGAGAGC	0.7147	0.0060	0.3562	0.1947	0.1615	intergenic_region	n.558720_558721insG	LDBPK_341310-LDBPK_341320			

Alternate Allele loss

Chr	Pos	REF	ALT	correlation	correlation_P	max	min	difference	Impact	Position_Change	Chromosome	NCBI ID	TriTrypDB ID	Annot
chr02	319334	G	C	-0.7437	0.0036	0.5443	0.1607	0.3836	synonymous_variant	c.1752G>C	p.Ser584Ser	CBZ31238	LdBPK_020680.1	ATP-dependent Clp protease subunit, heat shock protein 78 (HSP78), putative
chr34	1731924	C	T	-0.7143	0.0061	1.0000	0.7778	0.2222	intergenic_region	n.1731924C>T	LDBPK_044340-LDBPK_044360			
chr35	361527	A	C	-0.8640	0.0001	0.4627	0.3571	0.1055	intergenic_region	n.361527A>C	LDBPK_350830-LDBPK_350840			
chr36	973528	T	C	-0.7465	0.0034	0.5390	0.3273	0.2117	synonymous_variant	c.216T>C	p.Phe72Phe	CBZ38690	LDBPK_362510	sterol 24-c-methyltransferase, putative
chr36	2537912	G	A	-0.7454	0.0035	0.2275	0.1076	0.1199	intergenic_region	n.2537912G>A	LDBPK_366760-LDBPK_366770			
chr08	491302	GCACACACACACACACACACACACCACAA	GCACACACACACACACACACACCACAA	-0.7149	0.0060	0.6415	0.3273	0.3142	intergenic_region	n.491326_491327delAC	LDBPK_081250-LDBPK_081290			

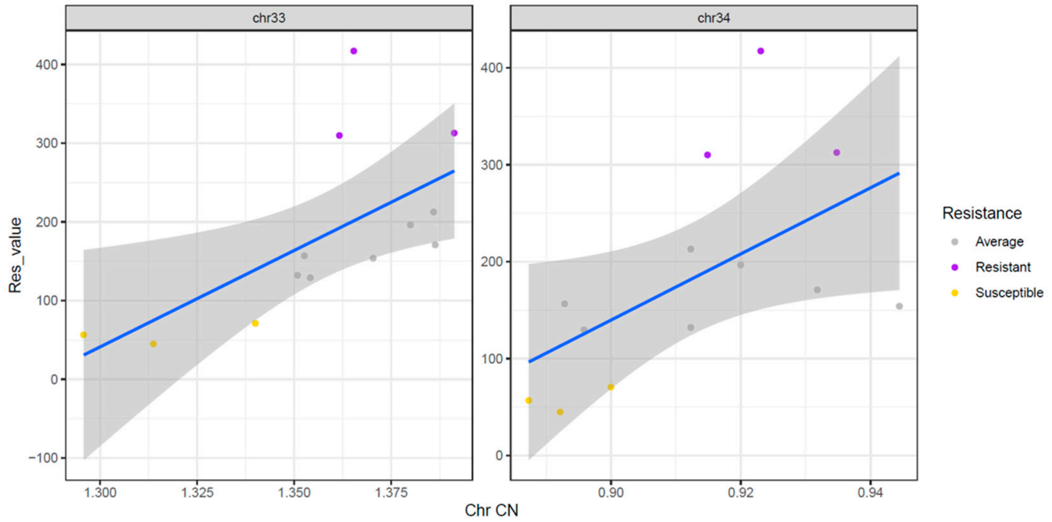

Figure S4. Correlation between CCNVs of Chr33 and Chr34 and PMM resistance: The box title contains the chromosome number. The colour of the dots reflects the level of resistance of the individual clones to PMM, where yellow, grey and purple correspond respectively to RI < 2; 2 < RI < 5 and RI > 5. Chr33 (r = 0.64, p-value = 0.02); Chr34 (r= 0.56, p-value = 0.04).

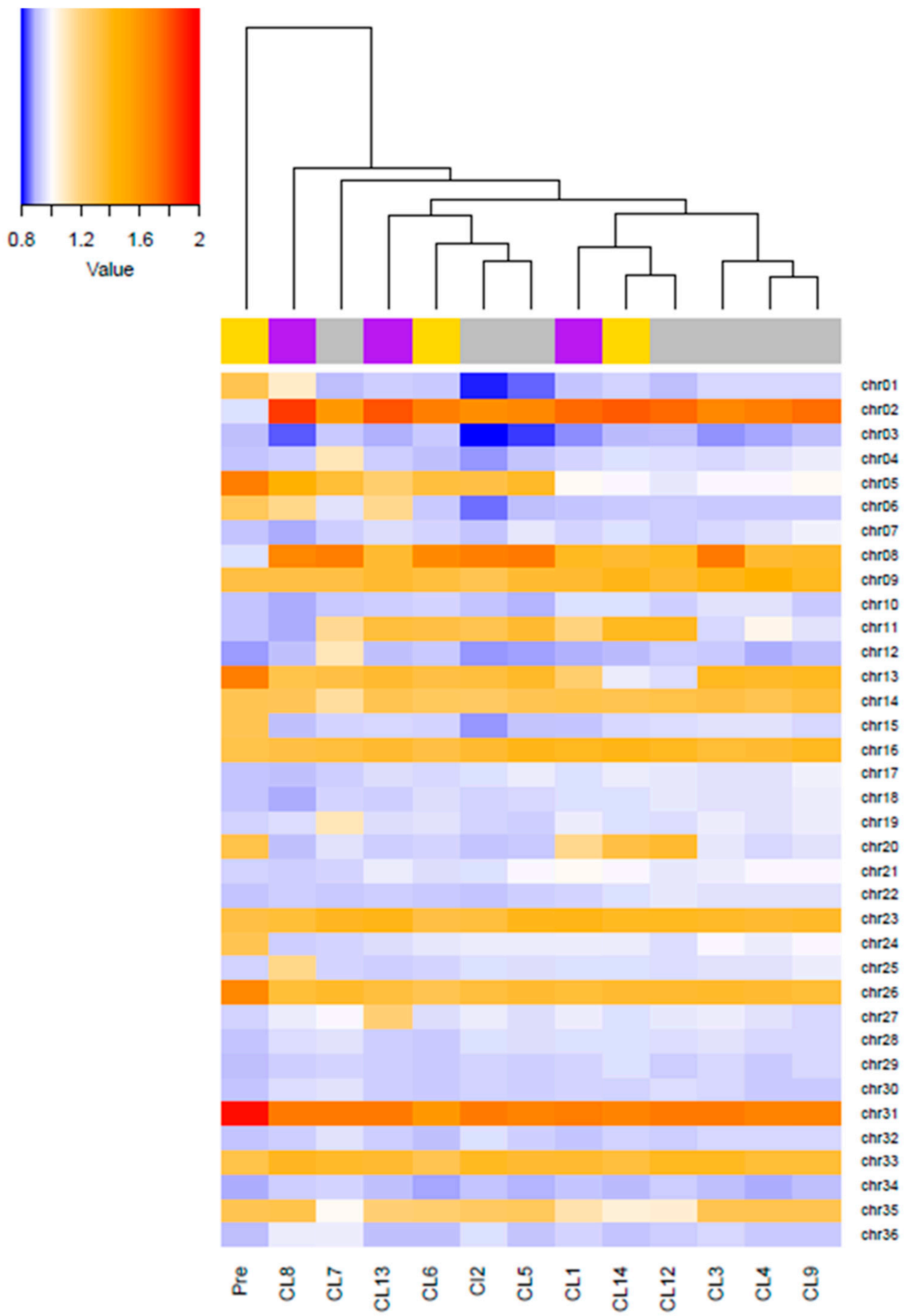

Figure S5: CCNV among the *L. donovani* isolates. The chromosomal somy estimation was performed based on the median coverage of non-outlier genes. In this heatmap, each line corresponds to a chromosome, ordered from 1 to 36; and each column corresponds to a *L. donovani* clone or the polyclonal population (Pre). The somy values are scaled to haploid genome copies, where a value of "1" corresponds to one chromosome copy per haploid genome copy. The somy values are represent by a colour ramp, from blue (low) to red (high) copies. The dendrogram on top represent the UPGMA clustering of the Manhattan distance of the chromosome copy numbers. The colour strip below the dendrogram represents the isolate PMM resistance, where RI < 2; 2 < RI < 5; and RI > 5 are represented respectively in yellow, grey and purple. The chromosomes did not cluster by PMM resistance values. The order of the isolates, from left to right, is: : "Pre", "CL 8", "CL 7", "CL 13", "CL 6", "CL 2", "CL 5", "CL 1", "CL 14", "CL 12", "CL 3", "CL 4" and "CL 9".

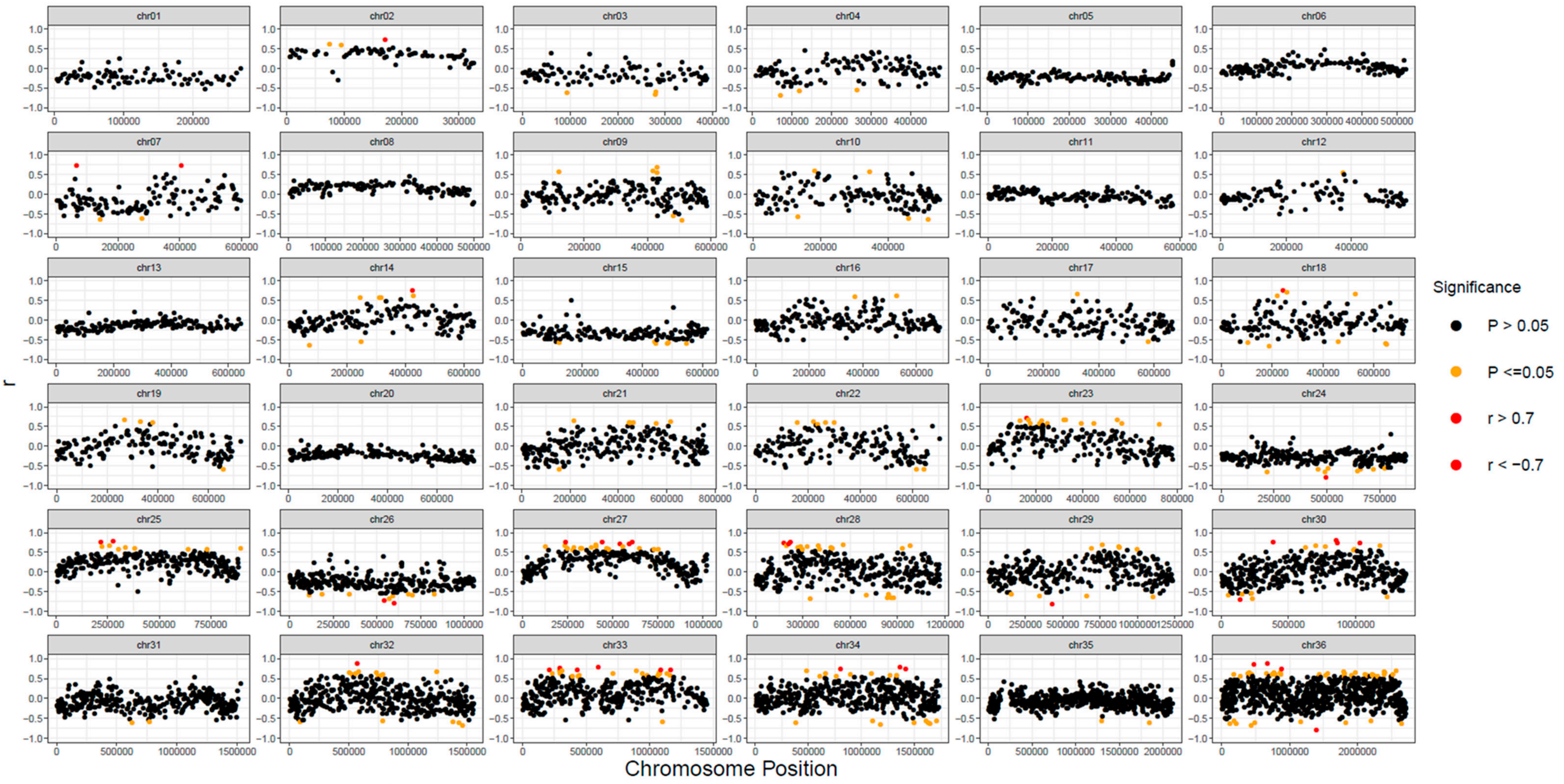

Figure S6: Correlation between Gene CNVs and PMM resistance in all *L. donovani* chromosomes. The X and Y axis correspond to, respectively, the chromosome position of each gene and its correlation to PMM phenotype. Each dot represent one gene, where significant correlations are represented by orange dots. Genes with strong significant positive or negative correlations (r ≥ 0.7 and r ≤ -0.7) to PMM resistance are represented by red dots.
